# Co-production of gallic acid and a novel cell-associated tannase by a pigment-producing yeast, *Sporidiobolus ruineniae* A45.2

**DOI:** 10.1186/s12934-020-01353-w

**Published:** 2020-04-25

**Authors:** Apinun Kanpiengjai, Chartchai Khanongnuch, Saisamorn Lumyong, Dietmar Haltrich, Thu-Ha Nguyen, Suwapat Kittibunchakul

**Affiliations:** 1 Division of Biochemistry and Biochemical Technology, Department of Chemistry, Faculty of Science, Chiang Mai University, Chiang Mai, 50200 Thailand; 2 Division of Biotechnology, Faculty of Agro-Industry, Chiang Mai University, Chiang Mai, 50100 Thailand; 3 Research Center for Multidisciplinary Approaches to Miang, Chiang Mai University, Chiang Mai, 50200 Thailand; 4 Department of Biology, Faculty of Science, Chiang Mai University, Chiang Mai, 50200 Thailand; 5 Food Biotechnology Laboratory, Faculty of Food Science and Technology, BOKU University of Natural Resources and Life Sciences, 1190 Vienna, Austria; 6 Institute of Nutrition, Mahidol University, 999 Phutthamonthon 4 Rd., Nakhon Pathom, 73170 Thailand

**Keywords:** Tannase, Tannins, Gallic acid, Yeast, *Sporidiobolus ruineniae*, Miang

## Abstract

**Background:**

Gallic acid has received a significant amount of interest for its biological properties. Thus, there have been recent attempts to apply this substance in various industries and in particular the feed industry. As opposed to yeasts, fungi and bacteria and their tannases have been well documented for their potential bioconversion and specifically for the biotransformation of tannic acid to gallic acid. In this research, *Sporidiobolus ruineniae* A45.2 is introduced as a newly pigment-producing and tannase-producing yeast that has gained great interest for its use as an additive in animal feed. However, there is a lack of information on the efficacy of gallic acid production from tannic acid and the relevant tannase properties. The objective of this research study is to optimize the medium composition and conditions for the co-production of gallic acid from tannic acid and tannase with a focus on developing an integrated production strategy for its application as a feed additive.

**Results:**

Tannase produced by *S. ruineniae* A45.2 has been classified as a cell-associated tannase (CAT). Co-production of gallic acid obtained from tannic acid and CAT by *S. ruineniae* A45.2 was optimized using response surface methodology and then validated with the synthesis of 11.2 g/L gallic acid from 12.3 g/L tannic acid and the production of 31.1 mU/mL CAT after 48 h of cultivation in a 1-L stirred tank fermenter. Tannase was isolated from the cell wall, purified and characterized in comparison with its native form (CAT). The purified enzyme (PT) revealed the same range of pH and temperature optima (pH 7) as CAT but was distinctively less stable. Specifically, CAT was stable at up to 70 °C for 60 min, and active under its optimal conditions (40 °C) at up to 8 runs.

**Conclusion:**

Co-production of gallic acid and CAT is considered an integrated and green production strategy. *S. ruineniae* biomass could be promoted as an alternative source of carotenoids and tannase. Thus, the biomass, in combination with gallic acid that was formed in the fermentation medium, could be directly used as a feed additive. On the other hand, gallic acid could be isolated and purified for food and pharmaceutical applications. This paper is the first of its kind to report that the CAT obtained from yeast can be resistant to high temperatures of up to 70 °C.

## Background

Gallic acid is a chemical constituent of the tannic acid molecules that are commonly found in tea leaves [1]. It has mainly been used in the pharmaceutical industry for the production of trimethoprim, an antibacterial agent, and gallate esters, that are used as preservatives in the food production industry [2]. However, gallic acid has received a significant amount of interest in terms of its biological properties, particularly for its antioxidant, antibacterial, anticarcinogenic, antiallergic and anti-inflammatory activities [3, 4]. The supplementation of gallic acid into broiler chick feed has improved the performance and jejunum intestinal morphology of broiler chicks [5]. Recent reports have revealed that proper minimum concentrations of gallic acid exhibit antimicrobial activity against human and animal pathogenic bacteria including *Escherichia coli*, *Pseudomonas aeruginosa*, *Staphylococcus aureus*, *Listeria monocytogenes* [6], and especially *Campylobacter jejuni* and *C. coli* [7]. Conventionally, acid hydrolysis of tannic acid is a method that is used to produce gallic acid, but it has some limitations in terms of its purity and yield [3]. Microbial and enzymatic conversions of tannic acid to gallic acid are attractive strategies for overcoming the drawbacks of the chemical method. Tannase catalyzes the hydrolysis of tannic acid to release glucose and gallic acid. It is primarily produced by bacteria and fungi as opposed to yeasts, from which its production has rarely been reported. Only *Candida* sp. [8] and *Kluyveromyces marxianus* [9], have been verified in terms of the ability to express tannases and the properties of their enzymes, but there is very little information on the bioconversion of tannic acid to gallic acid.

Animal feed additives are in fact one of the most interesting applications of tannase and gallic acid apart from its potential pharmaceutical and food applications. The addition of tannase in feed could decrease the antinutritional effect of tannins, thereby improving the digestibility of the feed and the degree of mineral absorption in animals [10]. Supplementation of gallic acid in feed mixtures has been reported to have significantly positive effects on the microbial community of animals [6, 7]. In a previous research, *Sporidiobolus ruineniae* A45.2 was found to be one of the most promising tannin-tolerant and tannase-producing yeasts that was isolated from Miang, a fermented tea-leaf prevalent in northern Thailand [11]. Among its physiological and biochemical characteristics is the formation of a red pigment. Consequently, it is assumed to be a member of the carotenoid family as has been reported in previous studies [12, 13]. Thus, *S. ruineniae* could be used in the production of gallic acid and biomass containing tannase activity and carotenoids, the latter having recognized health-promoting effects for both human and animals. The objective of this research study is to optimize the medium composition and conditions for the co-production of gallic acid obtained from tannic acid and tannase with a focus on developing an integrated production strategy for its applications as a feed additive. As this is the first report on tannase obtained from pigment-producing yeast, enzyme production and biochemical characterization were also included in this research study in order to evaluate its potential for further use.

## Results

### Bioconversion of tannic acid to gallic acid and tannase production by *S. ruineniae* A45.2

**Fig. 1:**
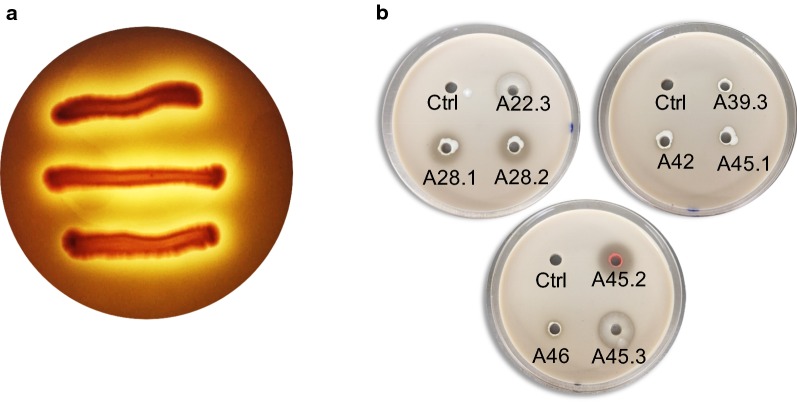
Growth of *S. ruineniae* A45.2 on YMA supplemented with 10 g/L tannic acid (**a**) and clear zone formation of nine tannin-tolerant yeast isolates on YMA supplemented with 10 g/L tannic acid (**b**) after culturing at 30 °C for 3 days

*Sporidiobolus ruineniae* A45.2 formed a clear zone around a red colony when grown on yeast malt agar (YMA) supplemented with tannic acid (Fig. 1). No tannase activity was detected in the culture medium when the yeast was cultivated in yeast malt broth (YMB) that was supplemented with 5 g/L tannic acid, but 5.2 ± 0.2 g/L gallic acid was satisfactorily obtained. Evaluation of enzyme activity using a whole cell as a biocatalyst revealed activity of 0.5 ± 0.06 and 1.3 ± 0.1 mU/mL after cultivation for 12 h and 24 h, respectively (Table 1). The amount of activity detected in the soluble fraction was proportional to 50% of the whole cell implying that cell disruption could partly release enzymes from the cell wall. Here, tannase produced by *S. ruineniae* A45.2 was designated as a cell-associated tannase (CAT). However, enhancement of its co-production capabilities is of high relevance with regard to the need to establish the highest level of bioconversion tannic acid to gallic acid and CAT yields. As CAT is a cell surface-displayed enzyme, production of CAT may be associated with cell numbers. In statistical optimization, gallic acid, CAT and cell numbers are considered response variables of the co-production system.

**Table 1:** Tannase activity, gallic acid content and viable cell numbers of *S. ruineniae* A45.2 when cultivated in YMB supplemented with 5 g/L tannic acid at 30 °C

Parameters	12 h	24 h
Cell free supernatant (mU/mL)	0	0
Soluble fraction (mU/mL)	0.25 ± 0.06	0.6 ± 0.12
Cell-associated tannase (mU/mL)	0.5 ± 0.06	1.3 ± 0.10
Gallic acid (g/L)	2.5 ± 0.1	5.2 ± 0.2
Viable cells (logCFU/mL)	6.95 ± 0.03	7.16 ± 0.02

### Optimization for co-production of gallic acid, CAT and viable cell numbers

Tannase produced by *S. ruineniae* A45.2 has been confirmed as an inducible enzyme that is naturally immobilized on the yeast cell surface (data not shown). Statistical optimization was used as a tool for the efficient co-production of gallic acid, CAT and cell number yields. Plackett and Burman design (PBD) was used to evaluate seven factors influencing the response variables as is shown in Table 2. Gallic acid content, CAT and cell numbers obtained from different compositions of media and incubation times based on the design matrix of PBD were assessed using analysis of variance (ANOVA) and the results are shown in Table 3. The data obtained from the experiment (gallic acid content, CAT, and cell numbers) were well-fitted with the least square linear regression model according to the significance of the model fit value with the R^2^-values and the adjusted R^2^-values being higher than 0.95. Tannic acid was the most significant variable that enhanced the production of gallic acid, CAT and viable cell numbers. Glucose had a significantly negative effect on the production of gallic acid and CAT, while it was positive in terms of viable cell numbers. Furthermore, there was a negative significant interaction between glucose and tannic acid that influenced cell numbers (data not shown). Basically, glucose is one of the most easily assimilated carbon sources for microbial growth. Tannic acid contains 10 galloyl units surrounding a glucose center. Therefore, it acts as an inducer for tannase production and a carbon source for cell growth. In the presence of tannic acid in the culture medium, sufficient supplementation of glucose seems to promote viable cell production rather than tannase and gallic acid productions. This is because yeast can more easily assimilate glucose in the culture medium than glucose that is derived from the degradation of tannic acid. Therefore, adequate amounts of glucose in the culture medium might be preferable to cell growth rather than tannase production. A reduction in enzyme production consequently affects gallic acid synthesis. Tannic acid was selected as the most significant variable that enhanced the co-production of gallic acid, CAT and cell numbers. In addition, glucose was selected in order to determine its effect on co-production, and at the same time to find an optimal concentration for the production process. Other variables were fixed at their low or high levels with regard to their effects and their relevant significant differences (10 g/L yeast extract, 2 g/L (NH_4_)_2_SO_4_, 0.5 g/L tween80 and 1 g/L glutamate, and 48 h cultivation time).

**Table 2:** Experimental design matrix of PBD and response variables for screening of the most significant factors affecting co-production of gallic acid, CAT and viable cell numbers

Run	A: Tannic acid (g/L)	B: Yeast (g/L)	C: Glucose (g/L)	D: (NH_4_)_2_SO_4_ (g/L)	E: Tween80 (g/L)	F: Glutamate (g/L)	G: Time (h)	Gallic acid (g/L)	CAT (mU/mL)	Viable cell numbers (logCFU/mL)
1	5	10	2	10	2	5	24	3.50	18.08	7.35
2	1	10	10	2	2	5	24	0.23	3.81	7.58
3	5	2	10	10	0.5	5	72	3.82	15.99	6.32
4	1	10	2	10	2	1	72	0.18	0.00	4.70
5	1	2	10	2	2	5	72	0.34	2.95	7.22
6	1	2	2	10	0.5	5	24	1.09	13.56	6.96
7	5	2	2	2	2	1	72	4.42	18.32	7.37
8	5	10	2	2	0.5	5	72	4.99	19.70	7.45
9	5	10	10	2	0.5	1	24	4.85	16.91	7.16
10	1	10	10	10	0.5	1	72	0.46	5.32	6.64
11	5	2	10	10	2	1	24	2.95	12.99	7.11
12	1	2	2	2	0.5	1	24	1.06	13.55	7.08
13	3	6	6	6	1.25	3	48	2.57	18.71	7.87
14	3	6	6	6	1.25	3	48	2.65	19.68	7.93
15	3	6	6	6	1.25	3	48	2.64	16.68	7.75

**Table 3:** Regression of coefficients and analysis of variance (ANOVA) of the first order model for response variables in PBD

Source	Gallic acid		CAT		Viable cell numbers	
	Coefficient	p-value	Coefficient	p-value	Coefficient	p-value
	Estimate	Prob > F	Estimate	Prob > F	Estimate	Prob > F
Model	2.32	< 0.0001	11.7641	0.0011	6.91	0.0006
A-Tannic acid	1.76	< 0.0001*	5.2333	< 0.0001*	0.21	0.0047*
B-Yeast extract	0.045	0.5695	− 1.1269	0.0921	− 0.31	0.0015
C-Glucose	− 0.22	0.0273*	− 2.1026	0.0097*	0.09	0.0879*
D-(NH_4_)_2_SO_4_	− 0.32	0.0047	− 0.7756	0.2173	− 0.18	0.0137
E-Tween80	− 0.39	0.0020	− 2.4072	0.0052	− 0.24	0.0049
F-Glutamate	3.30E−03	0.9658	0.5835	0.3398	0.02	0.6723
G-Time	0.045	0.5695	− 1.3847	0.0491	− 0.51	0.0002*

Gallic acid production: R^2^ = 0.9823, Adj-R^2^ = 0.9541, %C.V. = 2.17, lack of fit = 0.0182

CAT production: R^2^ = 0.9904, Adj-R^2^ = 0.9737, %C.V. = 10.78, lack of fit = 0.2074

Viable cell production: R^2^ = 0.9564, Adj-R^2^ = 0.9056, %C.V. = 11.91, lack of fit = 0.0019

* Significant difference at *p* < 0.05

In central composite design (CCD) optimization, the ANOVA results revealed that tannic acid (A) at a broader range of concentrations than that of PBD had a significant effect on gallic acid production, CAT and cell numbers, while glucose (B) showed insignificantly positive effects on all response variables. Tannic acid was a positive significant factor for gallic acid production due to the fact that it is the dependent factor for gallic acid production. However, it was a negative factor for cell and CAT production, explaining that at high concentrations of tannic acid, the growth of *S. ruineniae* was partially inhibited and enzyme production was decreased. Tannic acid is generally considered to be a microbial inhibitor. Although *S. ruineniae* is classified as a tannin-tolerant yeast that is similar to other microorganisms, particularly filamentous fungi, excessively high concentrations of tannic acid could be a factor in inhibiting its growth. The growth of *Aureobasidium pullulans* DBS66 was maximized when cultured in basal medium containing 10 g/L tannic acid and dramatically decreased in culture containing 20 g/L tannic acid [14]. This outcome was also observed for tannase production by *Bacillus licheniformis* [15]. Low production yields of tannase under high tannic acid concentrations can be explained in terms of tannase synthesis. Because of the deposition of gallic acid on the cell surface, increases in tannic acid concentrations induced an increase in the tannase synthesis of *Aspergillus awamori* followed by an eventual decrease [16]. Additionally, tannic acid–glucose interaction (AB) was significant at a *p*-value of less than 0.25. The second-order equations for co-production of gallic acid, CAT and viable cell numbers are given as follows:

$${\text{Gallic acid (g/L)}} = - 6. 6 1 1 2 + 2 . 2 4 2 9 {\text{A}} + 0 . 7 1 9 4 {\text{B}} - 0. 0 0 2 3 {\text{AB}} - 0. 0 7 3 1 {\text{A}}^{ 2} - 0. 0 3 2 2 {\text{B}}^{ 2}$$

$${\text{CAT (mU/mL)}} = 21 . 8 2 5 2 + 1 . 0 4 1 1 {\text{A }} + 2. 5 1 9 6 {\text{B }} + 0. 0 5 7 4 {\text{AB}} - 0. 1 0 5 9 {\text{A}}^{ 2} - 0. 2 3 1 9 {\text{B}}^{ 2}$$

$${\text{Cell numbers (logCFU/mL) }} = 7. 8 6 9 2 + 0. 0 9 8 5 {\text{A }} - 0. 0 3 4 0 {\text{B}} + 0. 0 0 3 4 {\text{AB}} - 0. 0 0 8 5 {\text{A}}^{ 2} - 0. 0 0 0 7 {\text{B}}^{ 2}$$

**Fig. 2:**
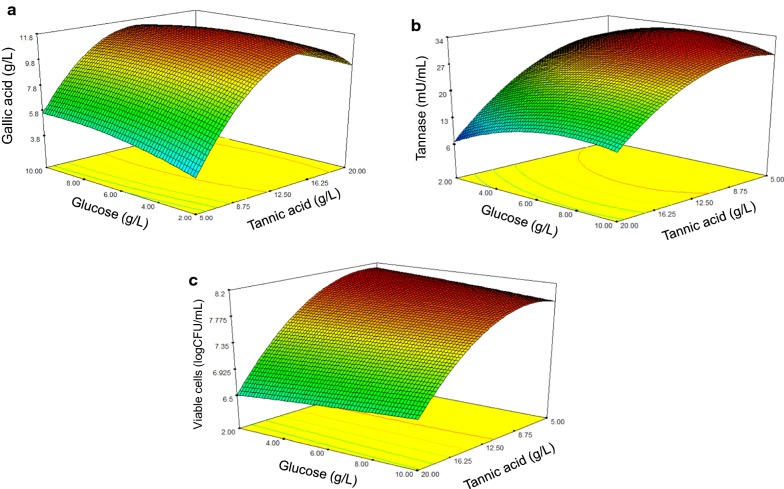
Three-dimensional curves and contour plots demonstrating the effect of glucose and tannic acid on gallic acid production (**a**), tannase (**b**) and viable cells (**c**)

**Fig. 3:**
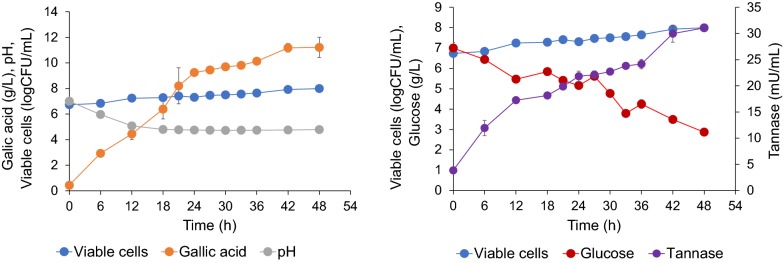
Time course of batch fermentation for co-production of gallic acid, CAT and viable cells using optimized medium and conditions by *S. ruineniae* A45.2 in 1-L fermenter

Regression models were employed to develop response surface plots as is shown in Fig. 2. These models produced acceptable results when *p *< 0.0001 with R^2^-values and adjusted R^2^-values between 0.92 and 0.97 (Table 4). This indicated that up to 92–97% of the variations in gallic acid content, CAT and viable cell number can be explained by theses equations. The predicted values of 11.46 g/L for gallic acid, 7.82 logCFU/mL for viable cells and 29.8 mU/mL (or 29.8 U/L) for CAT were predicted from the models and successfully validated at 97%, 96% and 96%, respectively when 12.32 g/L tannic acid and 6.91 g/L glucose were applied along with a considerable amount of other variables and conditions that have been previously described. Co-production in the 1-L fermenter (Fig. 3) confirmed that *S. ruineniae* A45.2 had potential in terms of the production of gallic acid, CAT and viable cell numbers due to maximal yields of 11.2 g/L gallic acid (equivalent to 91% conversion or 0.91 g gallic acid/g tannic acid), 31.1 mU/mL CAT and 7.99 logCFU/mL after 48 h of cultivation.

**Table 4:** Regression of coefficients and ANOVA of the second order polynomial model for response variables in CCD

Source	Gallic acid		CAT		Viable cell numbers	
	Coefficient	p-value	Coefficient	p-value	Coefficient	p-value
Model	11.49	< 0.0001	29.370	0.0001	7.804	< 0.0001
A-Tannic acid	2.12	0.0003	− 9.460	< 0.0001	− 0.698	< 0.0001
B-Glucose	0.21	0.5201	1.816	0.0962	0.003	0.9596
AB	− 0.67	0.1774	1.721	0.2388	0.104	0.2245
A^2^	− 4.11	< 0.0001	− 5.955	0.0006	− 0.478	< 0.0001
B^2^	− 0.52	0.1712	− 3.711	0.0081	− 0.011	0.8541
Lack of fit	0.0093		0.0025		0.0684	
R^2^	0.9654		0.9550		0.9703	
Adjusted R^2^	0.9406		0.9228		0.9491	
Predicted R^2^	0.7674		0.6891		0.8214

### Enzyme purification

**Fig. 4:**
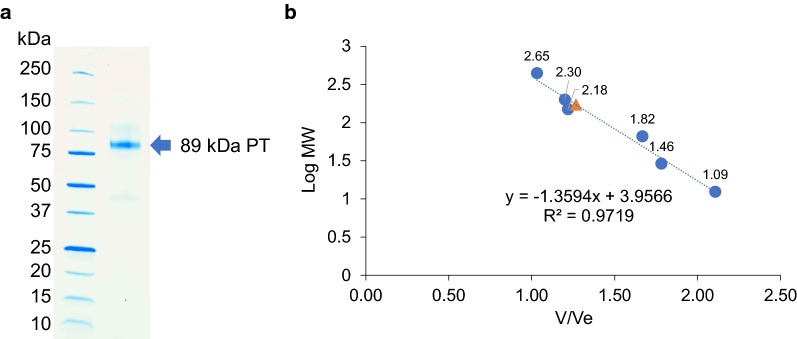
Molecular weight determination of tannase by SDS-PAGE (**a**) and gel filtration chromatography (**b**)

A total of 1950 mU of tannase equivalent to 381 mU/mg of protein was purified to homogeneity by applying the single-step process of Q-Sepharose anion-exchange chromatography. This yielded tannase that was purified 42.6-fold, with 62.3% recovery and a specific activity of 16,232 mU/mg. The sodium dodecyl sulfate polyacrylamide gel electrophoresis (SDS-PAGE) (Fig. 4a) revealed a single band of approximately 89 kDa, whereas the molecular weight estimated by gel filtration chromatography was found to be approximately 172 kDa (Fig. 4b).

### Characterization of purified tannase and CAT

**Fig. 5:**
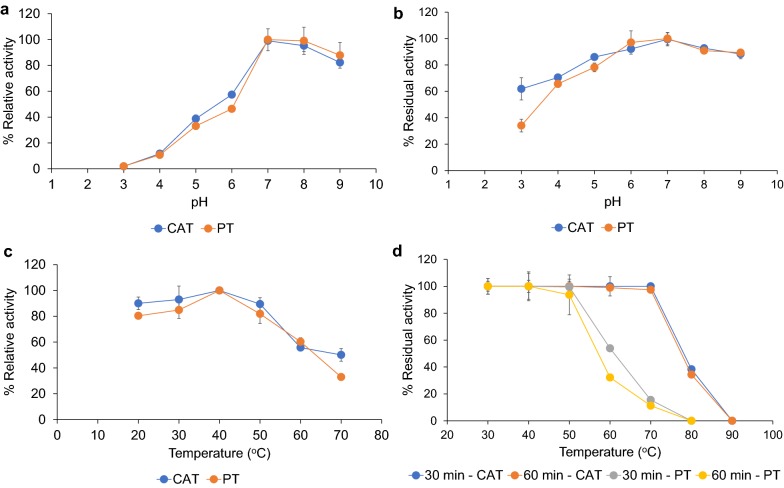
Effect of pH on tannase activity (**a**), stability (**b**). Effect of temperature on tannase activity (**c**) and stability (**d**)

Purified tannase (PT) was biochemically characterized in comparison with CAT in order to determine any distinctive differences. CAT was active at pH values ranging from 4.0 to 9.0 with an optimal pH of 7.0, while more than 80% relative activity was retained at pH values ranging from 8.0 to 9.0. These results were in accordance with those obtained from PT (Fig. 5a). For pH stability, both PT and CAT retained more than 80% of the original activity at pH values ranging from 5.0 to 9.0, while CAT was slightly more stable at pH 5.0 (Fig. 5b). The effects of temperature on enzyme activity and stability were determined at temperatures ranging from 20 to 90 °C. PT displayed the same range of optimal temperature as CAT since it showed the highest activity at 40 °C (Fig. 5c). In terms of thermostability, PT was stable at 20 to 50 °C for 60 min without any loss in the original activity, and its stability tended to decrease at temperatures above 50 °C. CAT was more stable than PT as it retained 100% residual activity after being incubated at 30–70 °C for 60 min (Fig. 5d).

Both PT and CAT were not affected by Na^+^, K^+^, Ca^2+^, Mg^2+^ and Mn^2+^, but they were partially inhibited by Cu^2+^ as is shown in Table 5. Under the same conditions, the relative measurement of the affinity of the methyl gallate revealed that PT and CAT exhibited similar *K*_m_ values of 2.6 ± 0.4 and 2.7 ± 0.4 mM and had *v*_max_ values of 583.5 ± 12.3 and 33.5 ± 2.31 (mU/mL), respectively. Thus, CAT could not only act in the same manner as PT but was also determined to be active with a better level of thermostability.

**Table 5:** Effect of various cations on CAT and PT activities

Cation (5 mM)	CAT	PT
Na	107.3 ± 6.3	100.1 ± 8.8
K	107.3 ± 6.3	99.8 ± 0.0
Cu	60.7 ± 0.0	63.3 ± 0.0
Ca	107.3 ± 6.3	91.2 ± 3.8
Mg	103 ± 5.5	98.1 ± 12.6
Mn	116 ± 3.5	107.1 ± 0.0
Control	100.0 ± 4.0	100.0 ± 4.6

### Repeatability of CAT

**Fig. 6:**
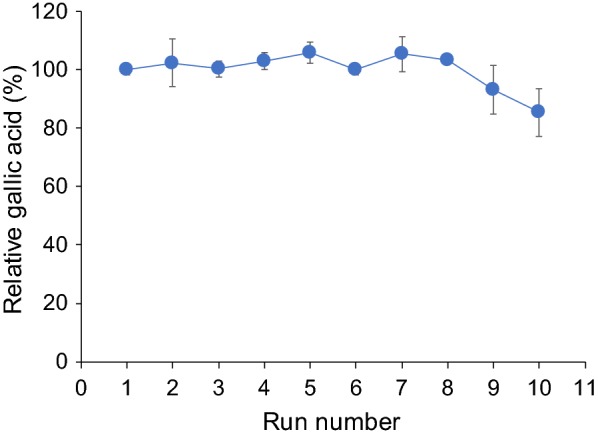
Operational stability of CAT under repeated use

CAT retained 100% relative gallic acid content after 8 runs of gallic acid production from methyl gallate under optimal conditions (40 °C, pH 7.0, and 30 min), while 80% relative gallic acid content was retained afterwards (Fig. 6).

## Discussion

*Sporidiobolus ruineniae* A45.2 is one of nine yeasts isolated from Miang that showed a positive result when using the visual reading method for detection of tannase activity in a previous study [11]. It was chosen for further characterization because it is a pigment-producing yeast, a characteristic that can be useful for feed application. As reported [17, 18], the tannase producing capacity of *S. ruineniae* may increase its value for feed applications. In addition, bioconversion of tannic acid to gallic acid by yeast sources poses a significant challenge as compared to bacterial or fungal biotransformation due to the lack of information regarding to such processes. In this research study, although CAT was expressed in terms of milliunits (mU), it clearly revealed a high degree of efficacy in terms of the bioconversion of tannic acid to gallic acid since a complete bioconversion of 5 g/L tannic acid to gallic acid was found within 24 h of cultivation. Enhancement of co-production by response surface methodology established the highest level of bioconversion, gallic acid production yields, viable cells and CAT in both a shake flask and a 1-L fermenter.

*Candida* sp. [8] and *K. marxianus* [9, 19] are the only two yeast species that are known to exhibit potential production of extracellular tannase; however, their potential for the bioconversion of tannic acid to gallic acid has not yet been evaluated. Although *Aspergillus* sp. was used for tannase production in several studies, gallic acid accumulation was not directly associated with its tannase yield, thus serving as only a tannase producer. An endogenous metabolism was a reason to explain the results [20, 21]. This outcome is similar to the findings of experiments involving *B. thurangiences* BN2 [22] and *Au. pullulans* [14]. In contrast with the findings of this study, gallic acid, CAT and viable cells were greatly accumulated in the fermentation process until the end of the cultivation period. In terms of the bioconversion of tannic acid to gallic acid, *A*. *niger* Aa-20 produced 7.64 g/L gallic acid from 12.5 g/L tannic acid with a maximum tannase activity 2479 U/L in the optimized fermentation process [23]. Bioconversion of tannic acid to gallic acid by *A. aculeatus* DBF9 was optimized with a yield of 6 g/L gallic acid from 30 g/L tannic acid [21]. Recently, anaerobic fermentation of *B. subtilis* AM1 and *Lactobacillus plantarum* CIR1 in 10 g/L tannic acid and under non-optimized conditions resulted in the maximum levels of tannase activity at 1400 and 1239 U/L and gallic acid contents of 2.41 and 2.37 g/L, respectively [24]. In addition, the aforementioned tannases are enzymes secreted to the fermentation medium. To the best of our knowledge, CAT has not yet been identified in yeast. Recently CAT was found in *Serratia facaria* [25, 26] and *B. massiliensis* [27], with activities corresponding to 9.65 and 0.56 mU/mL, respectively after optimization by RSM. The later strain of bacteria could enhance the formation of gallic acid from tea tannic acid with a maximum gallic acid production of approximately 0.475 g/L when cultured under optimized conditions [28]. Considering the yields of gallic acid and tannase, *S. ruineniae* clearly revealed a greater potential for gallic acid (11.8 g/L) and tannase (29.8 mU/mL) production.

*Sporidiobolus ruineniae* tannase was isolated from the cell wall, purified and biochemically characterized. This tannase has a molecular weight between 50 and 320 kDa, which is within the range of microbial tannases [29]. Based on the SDS-PAGE and gel filtration chromatography results, it is clearly suggested that CAT may be formed as a homodimeric enzyme with a molecular mass of approximately 180 kDa. Protein sequencing is required to assess *S. ruineniae* tannase identity. Depending on the type of microorganisms involved, tannases commonly consist of two or more units [29]. Recently, tannase obtained from *K. marxianus* was identified as having a monomeric state with a molecular weight of approximately 65 kDa after determination by SDS-PAGE [9], whereas tannase obtained from *Candida* sp. was a homodimeric enzyme with a molecular weight of 250 kDa [8].

With the exception of thermostability, CAT and PT showed similar activity profiles at different pH and temperature ranges. They also displayed a *K*_m_ value that is in agreement with that of other reported yeast tannases. CAT and PT were not affected by most cations used in this study as tannases do not generally require a cofactor for their activity [29]. The extracellular enzyme obtained from *K. marxianus* had an optimal pH value of 4.5 and was stable at pH values between 4.0 and 4.5 without any loss in activity after being incubated under these conditions at 30 °C for 30 min [9], while the optimal pH of tannase from *Candida* sp. was recovered at a pH of 6.0 [8]. These results were distinctively different from those of PT and CAT. Most fungal tannases were optimally active at a pH value of around 6.0, whereas bacterial tannases were mostly active at pH values between 7.0 and 9.0 [29–31]. Furthermore, fungal tannases were stable at a broader range of pH than their bacterial counterpart [29]. Although CAT and PT displayed the same range of optimal pH as the bacterial tannases, they were more stable since more than 80% of residual activities were retained from pH values ranging from 5.0 to 9.0. Notably, CAT was stable at up to 70 °C which was acknowledged as a broader range of temperature than PT and other reported yeast tannases [8, 9]. The resistance of CAT to irreversible denaturalization at 30–70 °C is possibly related to this tannase being embedded in the cell wall, which may prevent complete or partial unfolding. With regard to previously reported findings, microbial tannases generally have temperature optima in a range of 20–60 °C, and their thermostabilities are mainly between 30 and 60 °C. In addition, fungal tannases are more active and stable than the bacterial and yeast tannases under various temperature conditions [29, 30]. Since thermostability usually limits industrial applications of enzymes, the tannase that is associated with the cells used in this study could be an alternative choice.

The use of CAT as an immobilized enzyme is of particular interest as it is present on the cell wall surface and could be used repeatedly. Immobilization provides a number of advantages beyond the ability to separate products and recover enzymes for recycling and thus minimizing downstream processing costs [32]. It is expected that *S. ruineniae* and CAT could be used with regard to free and immobilized cells for the biotransformation of tannic acid to gallic acid, which could then be beneficial in various applications.

From a practical point of view, CAT obtained from *S. ruineniae* A45.2 would be suitable for use in the feed industry, in which the addition of tannase can help in terms of feed digestibility and absorption. However, feed additive enzymes must be thermostable in order to withstand relevant conditioning and pelleting temperatures [33]. Gallic acid that is formed in the fermentation medium can be directly applied as a feed additive, or isolated and purified for food and pharmaceutical applications. In addition, *S. ruineniae* biomass can be used as a source of carotenoids and tannase for feed application.

## Conclusions

This paper presents the first findings of CAT obtained from the pigment-producing yeast, *Sporidiobolus ruineniae*, which revealed a high degree of efficiency in terms of gallic acid production. It also exhibited viability in the presence of tannic acid after RSM optimization. CAT was more resistant to higher temperatures than the soluble tannase and exhibited similar biochemical properties. Consequently, it could be effectively employed in various applications and especially in the feed industry. The co-production of gallic acid, CAT and viable cells can be considered an integrated production strategy and can provide researchers with an opportunity to reduce costs associated with the process of downstream waste treatment.

## Methods

### Microorganisms and culture conditions

*Sporidiobolus ruineniae* A45.2 was grown on YMA (3 g/L yeast extract, 3 g/L malt extract, 5 g/L peptone, 10 g/L glucose and 20 g/L agar) that was supplemented with 5 g/L tannic acid (prepared in 8 g/L K_2_HPO_4_) at 30 °C for 48 h. Culturing was done in an Erlenmeyer flask at a temperature of 30 °C with shaking at 150-rpm. To prepare the seed inoculum, a loopful of *S. ruineniae* A45.2 was inoculated in YMB and incubated under the previously described culture conditions for 24 h or until the culture reached a maximal optical density at 600 nm (OD_600_) of 8–9.

### Assay of tannase and determination of gallic acid

Tannase and gallic acid content were assayed according to the method based on chromogen formation between gallic acid that was released from methyl gallate by a reaction of tannase and rhodanine (2-thio-ketothiazolidine) [34]. Briefly, the reaction mixture consisted of 50 µL of an appropriately diluted enzyme solution in 100 mM sodium-phosphate buffer pH 6.5 and in 50 µL of 12.5 mM methyl gallate in the same buffer. The reaction mixture was carried out at 30 °C and at 600 rpm for 10 min. To terminate the enzyme reaction and to determine gallic acid content, 60 µL of 0.667% (w/v) methanolic rhodanine solution was added to the mixture and it was left at room temperature (20 °C) for 5 min. Subsequently, 40 µL of 500 mM KOH was added to the mixture and it was left for 5 min. Finally, 800 µL of distilled water was added and the mixture was incubated at room temperature for 10 min prior to measuring the absorbance at 520 nm. One unit of tannase activity was defined as the amount of the enzyme releasing 1 µmole of gallic acid per minute under assay conditions.

### Evaluation of gallic acid production and tannase-producing ability by *S. ruineniae* A45.2

*Sporidiobolus ruineniae* A45.2 was grown on YMA supplemented with 5 g/L tannic acid and incubated at 30 °C. After 48 h of incubation, a clear zone formation was observed. To confirm and quantify the amount of tannase, 5 mL of seed inoculum was transferred to 25 mL of YMB supplemented with 5 g/L of tannic acid. It was then incubated at 30 °C with shaking at 150-rpm. At 12 h and 24 h of cultivation, samples were collected for the measurement of viable cells using the spread plate technique, while a portion was centrifuged at 6000 rpm at 4 °C for 20 min. The supernatant was used as a crude extracellular enzyme. On the other hand, the obtained cell pellet was washed twice with 20 mM sodium-phosphate buffer, pH 6.5 and resuspended in 10 mL of the same buffer. A half volume of the cell suspension was used as the whole cell tannase, while the rest was disrupted for 5 min using the Precellys 24 homogenizer (Bertin Technologies, France) in a bead beater. The resulting cell-free extract was designated as the soluble fraction. All fractions were assessed in terms of tannase activity by applying standard assay conditions.

### Optimization

PBD was used to screen and identify the most effective medium component and conditions for co-production of gallic acid and CAT. Based on the medium component that was reported for yeast tannase production [8] and live yeast cell production [35], a combination of seven factors including tannic acid, yeast extract, glucose, (NH_4_)_2_SO_4_, glutamate and tween80 were considered in preparation of the initial basal medium. Additionally, cultivation time was only considered a physical factor in the PBD experiment. Different media were prepared according to the design matrix of PBD (Table 2) that was created by Design Expert software version 7.0 (Stat-Ease Corporation, Minneapolis, USA). A total of 10% inoculum was transferred to the provided media and incubated under the standard culture conditions. Samples were collected in order to determine viable cell numbers, CAT and gallic acid content. ANOVA was used to evaluate the impact of a range of factors affecting the response variables. The factors whose *p*-values were less than 0.05 were considered significant factors and were further optimized by CCD.

In CCD, the five-level coded or actual values of each factor are represented by − α, − 1, 0, + 1, + α as is shown in Table 6, where − 1, + 1 correspond to the physical lower and upper limits of the explored factor space and − α, + α correspond to the new extreme physical lower and upper limits for all factors. Different medium compositions were varied based upon the CCD matrix. The experiment was conducted under conditions that have been previously described. Samples were collected to determine viable cell numbers, CAT and gallic acid content. The achieved values were analyzed by ANOVA and regression analysis, and fitted to the quadratic model equation as follows:

$${\text{Y}} =\upbeta_{0} + {{\sum\upbeta_{\text{i}}}} {{\text{x}}_{\text{i}}} + {{\sum\upbeta_{\text{ii}}}} {{\text{x}}_{\text{i}}^{2}} + {{\sum\upbeta_{\text{ij}}}} {{{\text{x}}_{\text{i}}}} {{{\text{x}}_{\text{j}}}}$$

where ${\text{Y}}$ is the predicted response variable; ${\text{x}}_{\text{i}}$ and ${\text{x}}_{\text{j}}$ are the independent variables of the experiment; $\upbeta_{0}$ is the intercept term; and $\upbeta_{\text{i}}$, $\upbeta_{\text{ii}}$ and $\upbeta_{\text{ij}}$ are the linear, squared and interaction coefficients, respectively. Finally, the regression equation together with the 3D-plots were used to predict the optimal values of the independent factors for the highest viable cell numbers and the highest levels of CAT and gallic acid production. Furthermore, validation of the predicted values was performed to ensure quadratic model fitting.

**Table 6:** Experimental design matrix of CCD and response variables for optimization of tannic acid and glucose concentrations

Run	A: Tannic acid (g/L)	B: Glucose (g/L)	Gallic acid (g/L)	CAT (mU/mL)	Viable cell numbers (logCFU/mL)
1	5	2	2.78	29.90	7.94
2	20	2	9.16	6.89	6.56
3	5	10	4.51	26.18	7.72
4	20	10	8.21	10.05	6.75
5	1.89	6	1.54	31.82	8.06
6	23.11	6	6.39	5.99	5.78
7	12.5	0.34	10.83	18.06	7.83
8	12.5	11.66	11.48	28.73	7.87
9	12.5	6	11.96	28.77	7.88
10	12.5	6	11.29	29.91	7.70
11	12.5	6	11.62	28.66	7.81
12	12.5	6	11.43	29.34	7.91
13	12.5	6	11.15	30.18	7.72

### Co-production of gallic acid and CAT in 1-L fermenter

To scale up the production of viable cells, CAT and gallic acid, the experiment was performed in a 1-L stirred tank fermenter (B.E. Marubishi Co Ltd., Tokyo, Japan) with a 60% working volume of the optimized medium. An inoculum of 10% (v/v) was transferred to the fermenter with an agitation speed of 250 rpm and an aeration rate of 0.2 vvm. The pH was not regulated during the cultivation process and the temperature was maintained at 30 °C. Samples were periodically collected in order to determine viable cells, CAT and gallic acid content.

### Preparation of CAT and enzyme purification

*Sporidiobolus ruineniae* A45.2 was grown in 1-L fermenter for 48 h according to the method previously described. Cell pellets were harvested by centrifugation at 8000×*g*, 4 °C for 20 min, washed twice with sodium phosphate buffer pH 6.0, resuspended in the same buffer and used as CAT for the purposes of characterization. For the purposes of enzyme purification, the cell suspension was supplemented with 0.5% (w/v) triton X-100 as the final concentration. Cell suspension was agitated at 4 °C and 50 rpm for 6 h before being centrifuged at 8000×*g*, 4 °C for 5 min. The clear supernatant was collected and used for enzyme purification. Free tannase was applied onto a 20 mL Q-sepharose^HP^ (GE Healthcare Bio-Sciences, Uppsala, Sweden) column that was equilibrated with 20 mM of sodium phosphate buffer at pH 7.0. The tannase was eluted by a linear gradient of 0–1000 mM of sodium chloride in 20 mM of sodium phosphate buffer pH 7.0 and with a flow rate of 0.5 mL/min. Active fractions were pooled and desalted using 10 kDa cut-off Amicon Ultra Centrifugal filter tubes (Millipore, Bileria, MA, USA).

### Determination of protein concentration

Protein concentrations were determined by the Bradford method using a protein assay system kit (BioRad, Hercules, CA, USA). Bovine serum albumin was used as a standard protein.

### Molecular weight determination

SDS-PAGE were performed using Mini-PROTEAN^®^ TGX Stain-Free Precast Gels (BioRad) according to the manufacturer’s instructions. Determination conditions were carried out by heating the protein solution to 100 °C for 4 min prior to being loaded onto the SDS-PAGE gel. Precision Plus Protein™ Standards (BioRad) was used as protein molecular mass markers. Protein bands were detected by being stained with Bio-Safe Coomassie (BioRad) according to the manufacturer’s instructions.

For gel filtration chromatography, the purified enzyme was loaded onto a glass column (60 × 1.3 cm) containing a Toyopearl HW-55 that was equilibrated with 20 mM sodium phosphate pH 7.0, and eluted with the same buffer at a flow rate of 0.25 mL/min. To calibrate the column, cytochrome *c* (12.4 kDa), carbonic anhydrase (29 kDa), bovine serum albumin (66 kDa), alcohol dehydrogenase (150 kDa), β-amylase (200 kDa) and ferritin (440 kDa) were used as the protein molecular weight standards. Blue dextran (2000 kDa) was used to determine the void volume (V_e_).

### Effect of pH and temperature on enzyme activity and stability

To determine the optimal pH values of PT and CAT, tannase activity (36 mU) was assayed under standard conditions over a pH range of 3.0–9.0. The buffers (100 mM) used were citrate–phosphate for pH values in a range of 3.0–5.0, sodium phosphate buffer for pH values in a range of 6.0–7.0 and Tris–HCl buffer for pH values in a range of 8.0–9.0. The pH stability of the enzyme was evaluated by incubating the enzyme at 37 °C at various pH values ranging from 3.0 to 9.0 in 20 mM of the appropriate buffers for 6 h. The activity without incubation was set to 100%. Residual activity was determined under standard assay conditions. The optimum temperature was determined at temperatures ranging from 20 to 70 °C under standard assay conditions. In terms of thermostability, the enzyme was pre-incubated at various temperatures ranging from 30 to 90 °C for 30 and 60 min. After that, it was placed on ice for 5 min prior to assaying the residual activity. The activity without incubation was set to 100%.

### Effect of cations

PT and CAT were assayed in the presence of 5 mM Na^+^, K^+^, Ca^2+^, Cu^2+^, Mg^2+^ and Mn^2+^ under otherwise standard assay conditions. Relative activities are provided and compared to those without cations.

### Determination of kinetic constants

The *K*_m_ and *v*_max_ values of PT and CAT were determined using various concentrations of methyl gallate from 0.25 to 12.5 mM. The assay conditions were otherwise identical to the standard assay conditions. The experimental data were fitted to the Michaelis–Menten equation using SigmaPlot version 12.0 (Systat software, Inc., San Jose, CA, USA).

### Operational stability

The operational stability of CAT was evaluated at its optimal pH value (pH 7.0) and temperature (40 °C) with an incubation time of 30 min in a repeated batch process. Biomass equivalent to 36 mU of tannase was incubated with 1 mL of 12.5 mM methyl gallate. After each operation, CAT was centrifuged at 5000×*g*, 4 °C for 5 min and was then washed with 20 mM of citrate–phosphate buffer pH 7.0. The resulting CAT was then subsequently used for another set of operations. Gallic acid production was determined for each batch.

## Data Availability

Not applicable.
